# Comorbidities in dementia during the last years of life: a register study of patterns and time differences in Finland

**DOI:** 10.1007/s40520-021-01867-2

**Published:** 2021-05-03

**Authors:** Saritha Susan Vargese, Pauliina Halonen, Jani Raitanen, Leena Forma, Marja Jylhä, Mari Aaltonen

**Affiliations:** 1 Faculty of Social Sciences (Health Sciences) and Gerontology Research Center (GEREC), University of Tampere, Tampere, Finland; 2 The UKK Institute for Health Promotion Research, Tampere, Finland; 3 Faculty of Social Sciences, University of Helsinki, Helsinki, Finland

**Keywords:** Dementia, Comorbidity, Morbidity patterns, Principal component analysis, Last years of life

## Abstract

**Background:**

Comorbidities have major implications for the care of people with dementia.

**Aim:**

To investigate the patterns of comorbidities in dementia in the last five years of life and how these patterns differed between three cohorts.

**Methods:**

The study included people who died at age 70 and above in 2001 (*n* = 13,717), 2007 (*n* = 34,750) and 2013 (*n* = 38,087) in Finland. ICD-10 morbidity data for a five-year period prior to death were extracted from national registers. Principal component analysis was employed to identify patterns for several morbidities. The associations of principal component scores with dementia were analysed using binary logistic regression. Linear regression was used to examine changes in the number of morbidities in patterns over time.

**Results:**

The morbidity patterns identified in the last years of life were (1) cardiometabolic disorders, (2) neurological, (3) cerebrovascular diseases and (4) musculoskeletal, thyroid and psychiatric disorders. Among the patterns, neurological and musculoskeletal, thyroid and psychiatric disorders were associated with dementia. The number of diagnoses in the cerebrovascular pattern increased and those in the musculoskeletal, thyroid and psychiatric pattern decreased over time.

**Discussion:**

Comorbidity patterns identified in this nationwide register study are largely in line with previous evidence. Time difference in these patterns provide crucial information for service planning.

**Conclusions:**

Comorbidities in dementia in the last years of life occur in patterns and change over time. More systematic monitoring and updated clinical guidelines are needed for the care of comorbidities with dementia.

## Introduction

Comorbidities among people with dementia is a rising concern for health care systems worldwide as the number of older adults continues to increase [1]. Research findings are contradictory: some studies report that people with dementia have a higher number of coexisting diseases [2, 3], some suggest that they are healthier [4], and some report similar levels of morbidity [5] as in people without dementia. The inconsistencies are likely to reflect the differences in studied populations and methods [5–8]. There is also a lack of evidence on how comorbidities in dementia change over time [9].

Other medical conditions in dementia might accelerate the decline of functional and cognitive abilities [10, 11]. Dementia is found to complicate the management of other illnesses [1, 12] and conversely, treatment of comorbid conditions may generate an opposite effect on the treatment of dementia due to improper drug combinations [4]. Furthermore, especially people living with advanced dementia may have difficulty communicating their health problems, resulting in hidden comorbidities, lack of treatment and poor health outcomes [7]. Therefore, dementia with coexisting diseases requires different treatment strategies. Multidisciplinary coordinated care has been recommended instead of a single disease approach [1].

A recent study reported that the incidence of dementia has declined by 13% in Europe [13] potentially explained by factors like improved living conditions, education, and more effective prevention and management of risk factors [14]. According to Alzheimer Europe report 2020, the prevalence of dementia has declined over the last decade (2008–2018), but the proportion of population having dementia is expected to double (1.57% in 2018 to 3% in 2050), because of the increase in ageing population, especially those above 85 years [15]. In Finland, the number of deaths from dementia has almost doubled in the last decade, and the country now has the highest age-adjusted dementia-related death rate in Europe [16]. Furthermore, as more people live in to very advanced age, the number of individuals with dementia is likely to grow [17, 18]. Increasing dementia is posing new requirements for care.

This study aims to identify the most common comorbidities and patterns of comorbidities in people with dementia in the last five years of life. Furthermore, it examines the differences between these patterns in 2001, 2007 and 2013. This information will help professionals redefine the necessary clinical guidelines [9] and develop nursing care on the continuum of services for better health outcomes and quality of life.

## Methods

The study population included three cohorts of people in Finland who died at age 70 and above in 2001 (*n* = 13,717), 2007 (*n* = 34,750) and 2013 (*n* = 38,087). The first dataset included a random sample of 40% of the 2001 cohort and the two other datasets comprised all decedents aged 70 or over in the respective years. The data included information on date and age of death and gender.

ICD-10 morbidity data for a five-year period prior to death were extracted from national registers as described below. A person was considered to have the diagnosis if it was indicated in any of the three source registers, namely The Causes of Death Register (Statistics Finland), The Care Register for Health Care and The Care Register for Social Welfare (Finnish Institute for Health and Welfare). These exhaustive registers cover every individual living in Finland and their use of health care, and the principle of registers have remained the same across study cohorts. Information on direct, immediate and underlying causes and other morbidities leading to death were extracted from the death register. The dementia group was identified using ICD-10 codes F00 (dementia in Alzheimer’s disease), F01 (vascular dementia), F02 (dementia in other diseases), F03 (unspecified dementia) or G30 (Alzheimer’s disease). In addition to dementia, the other diagnoses included in our analyses were hypertension (I10–I16), coronary artery disease (CAD) (I20–I25), diabetes (E10–E14), stroke (I60–I69), Parkinson’s disease or other neurological diseases (G00–G99 excluding G30), atherosclerosis or peripheral arterial occlusive disease (I65, I66, I67.2, I70, I73.9), cardiac insufficiency (I50), asthma or chronic obstructive pulmonary disease (COPD) (J40–J47), cancer (C00–C97), hip fracture (S72), thyroid disorders (E01–E07), diseases of lipoprotein metabolism and other lipidemias (E78), psychotic or neurotic disorders (F20–F29, F40–F48), depression (F32, F33), insomnia (F51, G47), joint arthrosis (M15–M19), osteoporosis (M80–M82) and renal insufficiency or failure (N17–N19). The list of morbidities was based on conditions found to be the most prevalent in the older population [7] and conditions associated with dementia in the previous literature [2, 12].

This study is part of the ongoing project: the longevity revolution: implications for the need and cost of health and social care (COCTEL) [19].

### Statistical analysis

The study participants’ characteristics were described as frequencies and percentages or means and standard deviations (SD). To determine the impact of dementia and time, differences between people with and without dementia and between the three cohorts were tested by independent samples *t* test for continuous variables and by Pearson’s Chi-squared test for categorical variables. A Mantel–Haenszel test was performed to investigate the trend in the proportion of people with and without dementia across the cohorts. We calculated the proportions of morbidities in the three cohorts of people with dementia, and then estimated odds ratios for each morbidity with cohort as the independent variable from binary logistic regression. Finally, we tested the linear trend of odds ratios for morbidities using the *contrast* command in Stata.

Principal component analysis (PCA) was used to identify morbidity patterns. PCA is a dimensionality reduction technique that was used to reduce the large number of diseases into a smaller number of components while retaining most of the relevant information in the large dataset. The number of components extracted depended on the eigenvalue (variances of the principal components) ≥ 1, scree plot (graphical plot of eigenvalues against component numbers) and clinically meaningful interpretable components. We proceeded with PCA as Bartlett’s test showed a significance of < 0.001. Component loadings > 0.3 were considered significant [20–23]. Chronic respiratory diseases had loadings > 0.3 in two components and negative loading in one component, and cancer was negatively loaded on two components and hence were excluded from PCA. Next, the associations of the principal component scores with dementia were examined by binary logistic regression analysis adjusted for age at death and gender. Finally, a linear regression model (adjusted for age at death and gender) with an interaction term between dementia and cohort was used to examine changes in the number of diseases in the morbidity patterns over time. As the number of diseases may have been affected by increasing age at death across the cohorts, we also run the analyses without age adjustment. Statistical analyses were performed using Stata 15 (College Station, TX, USA) and SPSS 25 (Armonk, NY, USA). We considered statistical significance at *p* < 0.05.

## Results

Almost one-third (31.7%) of the study population (*n* = 86,554) had dementia at the time of death. The figures for 2001, 2007 and 2013 were 24.5%, 30.2% and 35.6%, respectively. In each cohort, the share of women and mean age at death were significantly higher in people with dementia than in people without dementia. In people with dementia, the age and gender standardised mean number of morbidities increased significantly between the first two cohorts and then decreased slightly in the most recent cohort, still remaining at a higher level than in the first cohort (3.52, 3.77 and 3.74), while in people without dementia that number increased steadily (2.84, 3.08 and 3.20) (Table 1).

**Table 1:** Characteristics [percentage or mean with standard deviation (SD)] of the study population (*n* = 86,554): people above 70 years who died in 2001, 2007 and 2013

Characteristics	People with dementia				People without dementia			
	2001	2007	2013	*p* value	2001	2007	2013	*p* value
*n* (%)	3358 (24.5)	10,479 (30.2)	13,577 (35.6)	< 0.001^3a^	10,359 (75.5)	24,271 (69.8)	24,510 (64.4)	
Women (%)	69.0	66.4	65.4	< 0.001^3a^	55.5	53.2	51.6	< 0.001^3a^
Age at death (%)								
70–79	21.2	16.3	13.2		40.6	37.1	35.0	
80–89	50.7	52.7	51.0		42.9	44.5	44.4	
≥ 90	28.1	31.0	35.8		16.5	18.4	20.6	
*p* value^1^					< 0.001	< 0.001	< 0.001	
Age at death, mean (SD)	85.3 (6.4)	86.0 (6.4)	86.9 (6.3)		81.9 (7.0)	82.5 (7.2)	82.9 (7.4)	
*p* value^2^					< 0.001	< 0.001	< 0.001	
Mean number of diagnoses*								
Crude	3.53	3.77	3.74	< 0.001^3b^	2.82	3.08	3.21	< 0.001^3b^
Adjusted for age and gender	3.52	3.77	3.74	< 0.001^3b^	2.84	3.08	3.20	< 0.001^3b^
Level of comorbidity								
0	6.3	5.9	7.4		2.3	2.2	2.4	
1–2	46.9	41.3	40.5		44.2	38.1	35.2	
3–4	36.3	37.9	36.9		39.4	40.3	40.4	
≥ 5	10.5	14.9	15.2		14.1	19.4	22.1	
*p* value^1^					< 0.001	< 0.001	< 0.001	

*Dementia included

^1^Pearson’s Chi-squared test (people with and without dementia within year)

^2^Independent samples *t* test (people with and without dementia within year)

^3a^Mantel–Haenszel test for trend (within group)

^3b^From linear regression model (within group)

Table 2 reports the comorbidities for people with dementia over time. In the most recent year, the most prevalent comorbidities were coronary artery disease (37.3%), hypertension (36.0%), cardiac insufficiency (24.3%), and stroke (22.5%). Stroke, hip fracture, atherosclerosis, psychotic or neurotic disorders and depression decreased significantly across time while hypertension, cardiac insufficiency, diabetes, cancer, lipoprotein disorders, renal insufficiency, osteoporosis, insomnia and thyroid disorders increased. No significant cohort differences were found for coronary artery disease, Parkinson’s disease, or joint arthrosis.

**Table 2:** Proportions of people having different comorbidities with dementia in 2001, 2007 and 2013, differences between the years (odds ratios from binary regression models) and *p* values for time trend

Comorbidity	Proportions			Odds ratios (ref = 2001)		*p* for-trend of odds ratios
	2001	2007	2013	2007	2013	
CAD	38.8	44.2	37.3	1.25***	0.94	0.13
Hypertension	18.6	28.3	36.0	1.73***	2.47***	< 0.001
Cardiac insufficiency	21.0	24.9	24.3	1.25***	1.21***	< 0.001
Stroke	25.4	24.5	22.5	0.95	0.85***	< 0.001
Parkinson’s disease/neurological diseases	18.1	18.5	18.1	1.03	1.00	0.99
Diabetes	14.6	16.9	16.2	1.19**	1.14*	0.019
Hip fracture	16.6	15.7	14.0	0.93	0.81***	< 0.001
Cancer	11.6	13.5	14.4	1.19**	1.28***	< 0.001
Atherosclerosis	9.2	7.9	6.5	0.85*	0.69***	< 0.001
Asthma /COPD	7.4	9.0	9.1	1.22**	1.25**	0.001
Psychosis/ neurosis	4.3	3.3	2.6	0.77**	0.58***	< 0.001
Depression	5.7	4.0	2.8	0.69***	0.48***	< 0.001
Joint arthrosis	5.7	6.3	5.8	1.11	1.03	0.71
Thyroid disorders	2.7	3.0	3.4	1.12	1.28*	0.037
Renal insufficiency	2.2	4.1	7.1	1.89***	3.41***	< 0.001
Osteoporosis	2.0	4.0	4.5	2.02***	2.28***	< 0.001
Insomnia	0.5	0.8	1.3	1.55**	2.47***	< 0.001
Lipoprotein disorders	0.2	1.2	3.1	5.3***	13.2***	< 0.001

*CAD* coronary artery disease, *COPD* chronic obstructive pulmonary disease

*p < 0.05

**p < 0.01

***p < 0.001

PCA revealed four morbidity patterns in the whole sample (Table 3). The first component, cardiometabolic disorders*,* had heavy loadings (factor loading > 0.4) on cardiac insufficiency, coronary artery disease, renal insufficiency, diabetes and atherosclerosis or peripheral arterial occlusive disease. The second component was named as neurological diseases, with heavy loadings on insomnia and Parkinson’s disease or other neurological diseases. The third component was cerebrovascular diseases due to heavy loadings on hypertension, diseases of lipoprotein metabolism and stroke. The last component, musculoskeletal, thyroid and psychiatric disorders, included osteoporosis, hip fracture, joint arthrosis, thyroid disorders, depression and psychotic or neurotic disorders.

**Table 3:** Principal component loading matrix for morbidities representing the four morbidity patterns identified through principal component analysis

Morbidity	Component 1: Cardiometabolic disorders	Component 2: Neurological disorders	Component 3: Cerebrovascular diseases	Component 4: Musculoskeletal, thyroid and psychiatric disorders
Cardiac insufficiency	**0.78**	0.03	− 0.04	0.10
CAD	**0.63**	− 0.01	0.08	− 0.07
Renal insufficiency	**0.60**	− 0.04	0.08	− 0.02
Diabetes	**0.49**	0.19	0.36	− 0.14
Atherosclerosis	**0.35**	0.05	0.22	− 0.18
Insomnia	0.17	**0.89**	− 0.01	− 0.06
Parkinson’s disease/neurological diseases	− 0.14	**0.83**	0.14	0.06
Hypertension	0.30	0.10	**0.65**	0.19
Lipoprotein disorders	0.00	0.13	**0.64**	0.02
Stroke	− 0.14	0.23	**0.59**	− 0.07
Osteoporosis	0.06	− 0.15	0.02	**0.60**
Depression	− 0.04	0.26	0.04	**0.59**
Hip fracture	0.01	− 0.12	− 0.00	**0.49**
Thyroid disorders	0.02	− 0.04	0.20	**0.45**
Psychosis / neurotic disorders	− 0.10	0.22	− 0.16	**0.44**
Joint arthrosis	0.11	− 0.04	0.16	**0.34**

Bold indicates factor loadings of morbidities in the pattern

*CAD* coronary artery disease

Table 4 demonstrates the association between the principal component scores of the patterns identified and dementia (adjusted for age, gender and cohort). The odds of having dementia was higher for those whose principal scores for neurological diseases (OR_adj_ 1.79, 95% confidence interval (CI) 1.65–1.93) and musculoskeletal, thyroid and psychiatric disorders (OR_adj_ 1.78, 95% CI 1.64–1.92) were higher. Meanwhile, the odds of having dementia was lower for those whose principal scores for cardiometabolic disorders (OR_adj_ 0.34, 95% CI 0.32–0.35) and cerebrovascular diseases (OR_adj_ 0.71, 95% CI 0.67–0.74) were higher.

**Table 4:** Associations of principal component scores of morbidity patterns with dementia. Odds ratios, 95% confidence intervals and *p* values from logistic regression model

Variable	Odds ratio	95% confidence interval	*p* value
Cardiometabolic disorders*	0.34	0.32–0.35	< 0.001
Neurological disorders*	1.79	1.65–1.93	< 0.001
Cerebrovascular diseases*	0.71	0.67–0.74	< 0.001
Musculoskeletal, thyroid and psychiatric disorders*	1.78	1.64–1.92	< 0.001
Age at death	1.08	1.08–1.08	< 0.001
Gender (ref. male)	1.33	1.28–1.37	< 0.001
Cohort (ref. 2001)			
2007	1.35	1.29–1.42	< 0.001
2013	1.66	1.59–1.75	< 0.001

*Principal component score

**Fig. 1:**
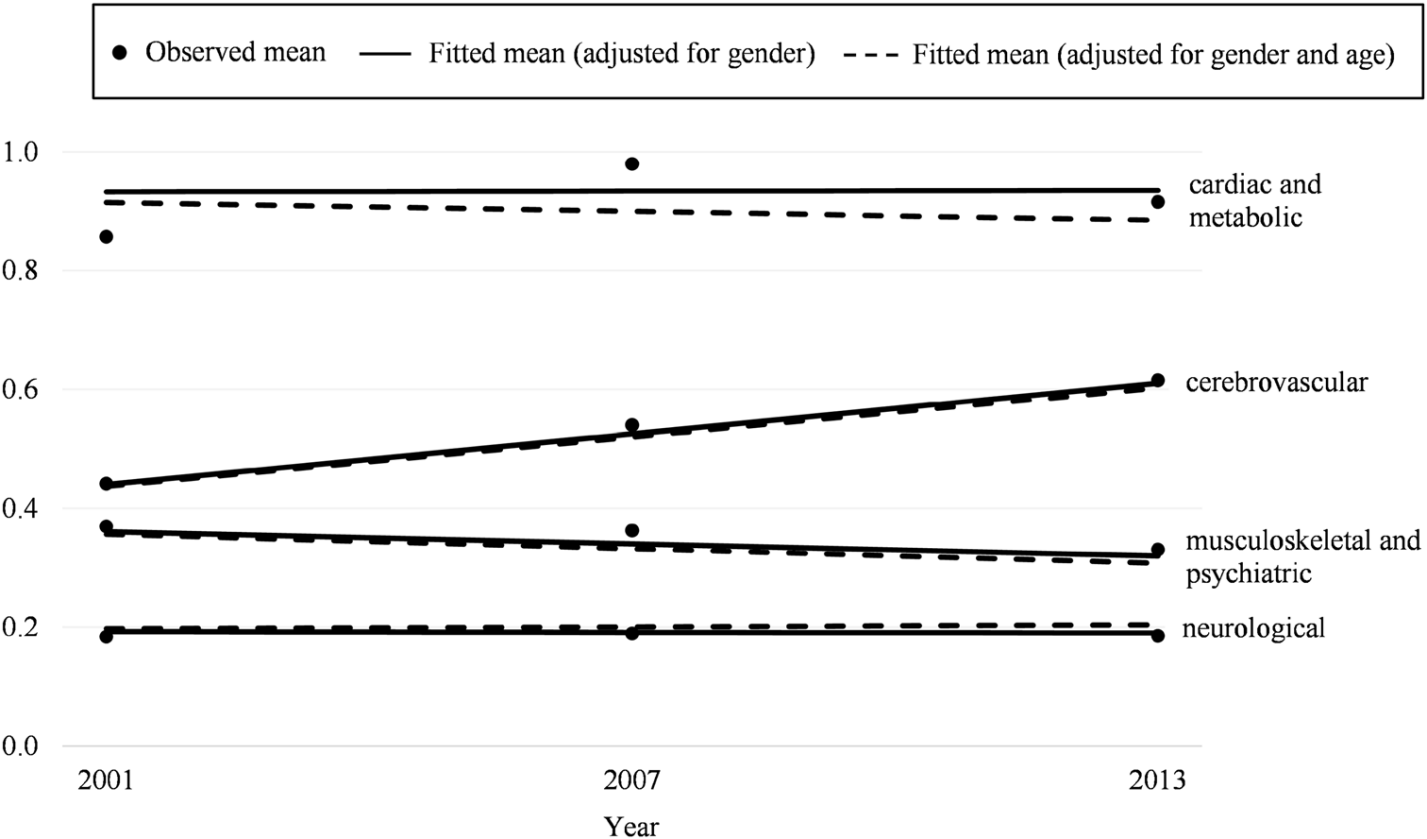
Mean number of diseases in the four morbidity patterns in the three cohorts. Trajectories from linear regression models, adjusted for gender (solid line) and additionally adjusted for age at death (dotted line)

Figure 1 shows the observed means in the three cohorts and fitted trajectories for changes in the number of diseases in the morbidity patterns over time in people with dementia from the linear regression model. There was a significant increase in cerebrovascular diseases (*p* < 0.001) and a decrease in musculoskeletal, thyroid and psychiatric disorders (*p* < 0.001). The decreases in cardiometabolic disorders (*p* = 0.11) and neurological and lung-related diseases (*p* = 0.32) were not statistically significant. Cardiometabolic disorders was the only disease pattern that showed a different time trend depending on whether the changing age structure was taken into account. Without age adjustment, there was a slight increase in cardiometabolic diagnoses but that turned to decline when adjusted for age.

## Discussion

We found mixed time trends for comorbidities in the last years of life of people with dementia: some increased, a few decreased and some were stable between the cohorts. The most frequent conditions in people with dementia were coronary artery disease followed by hypertension, cardiac insufficiency and stroke. Our findings are supported by previous research which has reported that hypertension [1, 2], cardiovascular diseases, stroke and diabetes are the most common diseases among people with dementia [12].

### Changes in comorbid conditions over time

Trends in the chronic disease burden in the older population have been quite extensively explored [24, 25], but to our knowledge, there are no previous studies on how comorbidities in people with dementia change over time. Of the diagnoses, hypertension, cancer, renal insufficiency, osteoporosis, insomnia and lipoprotein disorders became more frequent in people with dementia. The increasing prevalence of hypertension may be due to lowered blood pressure cut-offs for diagnosis [26]. Diabetes has increased over time, which may explain the increasing occurrence of renal insufficiency in the sample. Among cardiovascular diseases, cardiac insufficiency increased while coronary artery diseases showed no change in people with dementia. An earlier Finnish study reported a decrease in cardiovascular diseases in working-age people due to a reduction in risk factors and a shift to older age due to better treatment and survival [27]. An increasing trend in the prevalence of comorbidities may partly be due to increasing life expectancy, changing diagnostic criteria, more active diagnosing of dementia and some of the comorbidities, and the availability of more effective drugs [25]. The prevalence of comorbidities varies with the type of dementia and increases with severity of cognitive impairment [2, 8].

### Comorbidity patterns and their associations with dementia

A better understanding of conditions that coexist with dementia in specific patterns is crucial for care planning [11]. Using PCA, a novel way to summarise complex conditions, we were able to identify four morbidity patterns, two of which were associated with dementia. The coexistence of neurological pattern with dementia represents a clinically interpretable pattern and is supported by previous studies [28–30]. Insomnia is found to be associated with increased risk of dementia and is one of the most common symptoms in Parkinson’s disease [29], thus explaining the pattern.

Despite differences in methodological and statistical approaches, earlier studies have consistently identified cardiometabolic, mental health, musculoskeletal, and neuropsychiatric patterns in adults [6, 23]. Such patterns might stem from similar risk factors or common pathophysiological mechanisms [9]. The diseases in the fourth pattern we identified—musculoskeletal, thyroid and psychiatric disorders—are interrelated: it has been reported that people on medications for depression or psychosis, with adverse effects such as sedation and hypotension, are at greater risk for falls and fractures [31]. There are several reports of a significant association between depression and osteoporosis [32] and hypothyroidism and depression [33], lending support to the convergence thesis. The evidence suggests associations between dementia and depression [7], hip fracture [31], higher thyroid function [34] and osteoporosis [35]. These associations underline the need for early diagnosis and management of depression and osteoporosis as well as the importance of identifying the type of thyroid disorders associated with dementia. Our results are in line with existing reports on disease patterns coexisting with dementia in older adults, including anxiety, depression, somatoform disorders and pain, and neuropsychiatric disorders [2, 28]. The morbidity pattern showing the greatest increase over time was that of cerebrovascular diseases, a group not specifically associated with dementia. Among the patterns that were more frequent in dementia patients than in those without dementia, musculoskeletal, thyroid and psychiatric disorders showed a significant decline between the cohorts. The decrease in this pattern is attributable to the decrease in several diagnoses associated with this pattern, namely hip fracture, psychotic or neurotic disorders and depression, while osteoporosis and thyroid disorders increased.

This first nationwide study in Finland provides information on changes over time in comorbidity in dementia and introduces a novel approach to comorbidity research by employing PCA to detect concurrent diagnoses in dementia. It overcomes many limitations of earlier comorbidity studies, drawing its data from multiple national registers and using a comprehensive list of morbidities and disease patterns instead of any single disease [9]. The exhaustive registers contain information on every individual in Finland. Diagnosis of dementia (ICD10) is entered in the Care Register for Health Care or the Care Register for Social Welfare when a person with dementia is admitted to care, i.e., uses the service. In addition, the Causes of Death Register includes information on dementia diagnosis. These practices have not changed from 2001 to 2013, and we have no reason to suspect differences between the study years in this respect. It is important to note, however, that we can draw no inferences about causality between dementia and comorbidities and that the trends observed must be interpreted with caution as they largely depend on the population, clinical criteria used, hospital reporting, death registry data and health seeking patterns [5, 7]. People in the advanced stages of dementia may not be able to communicate their symptoms, which may lead to underestimation of several medical conditions. Furthermore, there may be differences in the associations of different subtypes of dementia, but our main concern here is with population health needs and care organisation rather than etiology. As the age structure and chronic conditions among old are approximately similar, we believe that largely our findings are applicable to Europe, especially Northern countries [36].

Our findings warrant the development of improved models of dementia care, both clinical and social, that anticipate the future scale of dementia and its comorbidities. New clinical guidelines are needed for coexisting diseases as most current guidelines are disease-specific [22], often leading to polypharmacy, drug interactions and iatrogenic events [3]. Coordination and communication between specialists and primary care providers may help to attain the best possible outcomes.

In conclusion, our study highlights the medical complexity associated with dementia, underscoring the importance of regular monitoring and an integrated treatment protocol with a multiple disease approach. This will help to improve the quality of life of people with dementia. Further research should focus on the mechanisms in the relationship between comorbidity patterns and dementia and its subtypes.

## Data Availability

The research group has received permission to use the register data from Statistics Finland and the National Institute for Health and Welfare. The register data are not owned by the research group. The data are confidential and not freely available.
